# Supplementary material for: Bacteriocin-Producing Lactic Acid Bacteria Strains with Antimicrobial Activity Screened from Bamei Pig Feces
Source: Foods. 2022 Feb 28;11(5):709. doi: 10.3390/foods11050709 (PMC8909009; doi:10.3390/foods11050709)
Supplement: Supplementary file 1 [file foods-11-00709-s001.zip › foods-1598942-supplementary.pdf]

Supplementary Material

Table S1. Identification results of 8 antibacterial active LAB strains

Strain	Origin	Shape	Gram	CAT	Glu	Identification
QP3-2	breeding boar	rod	+	-	-	*Lactiplantibacillus plantarum*
QP4-2	breeding boar	rod	+	-	-	*Lactiplantibacillus plantarum*
QP19-1	breeding boar	rod	+	-	-	*Lactiplantibacillus plantarum*
QP28-1	weaned piglets	rod	+	-	-	*Lactiplantibacillus plantarum*
QP20-2	breeding boar	coccus	+	-	-	*Lactococcus lactis*
QP22-2	weaned piglets	coccus	+	-	-	*Enterococcus hirae*
QP23-1	weaned piglets	coccus	+	-	-	*Enterococcus hirae*
QP33-2	unweaned piglets	rod	+	-	-	*Lactiplantibacillus plantarum*

Notes: Gram, CAT, and Glu represent the results of Gram reactions, catalase activity, and gas production from glucose, respectively; “+” represents positive and “-” represents negative.

Table S2. The wide-spectrum antibacterial activity of CFS of 8 LAB strains

Strain	*E.coli* ATCC 30105	*M. luteus* ATCC 4698	*Staph. aureus* ATCC 29213	*Ps. aeruginosa* ATCC 27853	*L. monocytogenes* BAA	*B. subtilis* ATCC 6633	*Salm. enterica* ATCC 43971	*Salm. enterica* ATCC 13076
QP3-2	++	++++	++	++	++++	+++	++	++
QP4-2	++	++++	++	++	++++	+++	++	++
QP19-1	++	++++	++	++	++++	+++	++	++
QP28-1	++	+++	++	++	++++	+++	++	++
QP20-2	+	+++	++	++	++	++	-	-
QP22-2	-	+	-	++	-	+	-	-
QP23-1	+	++	++	++	++	++	+	-
QP33-2	++	++++	++	+++	+++	++	++	++

Notes: the inhibition zone contains the external diameter of the cup（10 mm）. Diameter of the inhibition zone: -, no inhibition; +, 10-15 mm; ++, 15-20 mm; +++, 20–25 mm; ++++, more than 25 mm.

Table S3. The physiological and biochemical characteristics of 8 LAB strains.

Strain	Growth in NaCl		Growth at Temperature (°C)					Growth at pH								Fermentation type
	3.5	6.5	5	10	30	45	50	3	3.5	4	4.5	5	8	9	10	
QP3-2	++	++	+	+	++	+	-	-	+	++	++	++	++	++	++	Homo
QP4-2	++	++	w	+	++	+	w	-	+	++	++	++	++	++	++	Homo
QP19-1	++	++	+	+	++	+	-	-	w	++	++	++	++	++	+	Homo
QP28-1	++	++	w	+	++	+	w	-	+	+	++	++	++	++	+	Homo
QP20-2	+	-	-	w	++	-	-	-	-	-	-	w	+	w	w	Homo
QP22-2	+	+	-	w	++	+	-	-	-	++	w	w	+	w	+	Homo
QP23-1	+	+	-	w	++	+	w	-	-	w	w	+	+	+	+	Homo
QP33-2	++	++	w	+	++	+	w	-	w	+	++	++	++	+	+	Homo

Table S4. The bile salt tolerance of 8 LAB strains.

Strain	Time (hour)	Control	0.1%	0.2%	0.3%
QP3-2	0	8.14±0.01	8.01±0.08	6.86±0.01	5.75±0.06
	1	8.02±0.04	8.06±0.15	6.73±0.06	5.33±0.06
	2	8.49±0.01	8.07±0.04	6.83±0.04	5.62±0.06
	3	8.67±0.06	8.19±0.01	6.81±0.06	4.89±0.03
	4	8.91±0.04	8.27±0.10	6.92±0.03	5.02±0.04
QP4-2	0	8.14±0.12	8.06±0.14	6.75±0.04	4.84±0.09
	1	8.06±0.14	7.92±0.08	8.28±0.03	4.47±0.02
	2	8.48±0.06	8.13±0.05	6.72±0.08	4.47±0.02
	3	8.68±0.07	8.23±0.02	6.68±0.03	4.50±0.08
	4	8.84±0.05	8.12±0.04	6.78±0.05	4.47±0.04
QP19-1	0	8.18±0.03	8.14±0.19	5.91±0.09	3.92±0.03
	1	8.10±0.05	8.01±0.03	4.90±0.04	3.13±0.07
	2	8.46±0.02	8.03±0.04	4.82±0.13	2.96±0.24
	3	8.71±0.03	8.16±0.09	4.78±0.05	2.90±0.35
	4	8.84±0.09	8.19±0.02	4.86±0.11	ND
QP28-1	0	8.18±0.07	8.02±0.04	5.32±0.15	3.65±0.09
	1	8.23±0.06	7.98±0.14	4.97±0.04	2.90±0.17
	2	8.54±0.07	7.85±0.09	4.95±0.01	3.10±0.17
	3	8.94±0.03	8.51±0.07	4.85±0.02	2.80±0.17
	4	8.91±0.03	8.69±0.08	4.77±0.06	ND
QP23-1	0	7.69±0.19	7.46±0.40	4.98±0.03	ND
	1	7.92±0.16	7.39±0.09	3.66±0.10	ND
	2	8.42±0.56	7.13±0.08	3.48±0.18	ND
	3	8.17±0.02	7.03±0.05	3.42±0.10	ND
	4	8.26±0.06	7.00±0.04	3.28±0.17	ND
QP33-2	0	8.22±0.06	7.67±0.07	5.73±0.10	4.51±0.03
	1	8.23±0.11	7.63±0.10	5.11±0.03	3.93±0.06
	2	8.59±0.07	8.08±0.05	4.96±0.03	3.46±0.15
	3	8.83±0.05	8.63±0.03	4.91±0.17	3.42±0.05
	4	9.19±0.03	8.68±0.09	4.70±0.04	3.60±0.05

Notes: the value is the number of viable LAB counts (log CFU/mL) under different bile salt concentration conditions.

Table S5. The antibiotic susceptibility of 8 LAB strains.

	AMP	E	CT	P	CIP	CN	C	VA	RD	TE
QP3-2	S	S	R	R	R	R	S	R	MS	S
QP4-2	S	S	R	R	R	R	S	R	MS	S
QP19-1	S	S	R	R	R	R	S	R	MS	S
QP28-1	S	R	R	R	R	R	MS	R	MS	R
QP20-2	S	S	R	R	R	S	S	MS	R	S
QP22-2	S	MS	R	R	R	R	R	S	S	R
QP23-1	S	S	R	MS	R	R	S	MS	MS	S
QP33-2	S	R	R	R	R	R	MS	R	MS	R

Notes: (a) the antibiotics used in this study are presented as follows: AMP (ampicillin, 10 μg/disk); E (erythromycin, 15 μg/disk); CT (colistin sulphate, 10 μg/disk); P (penicillin, 10 μg/disk); CIP (ciprofloxacin, 5 μg/disk); CN (gentamicin, 10 μg/disk); C (chloramphenicol, 30μg/disk); VA (vancomycin, 30μg/disk); RD (rifampicin, 5 μg/disk); TE (tetracycline, 30 μg/disk).

(b) The antibiotic susceptibility of the strains was scored as R=resistant, MS=moderate resistance, and S=susceptible

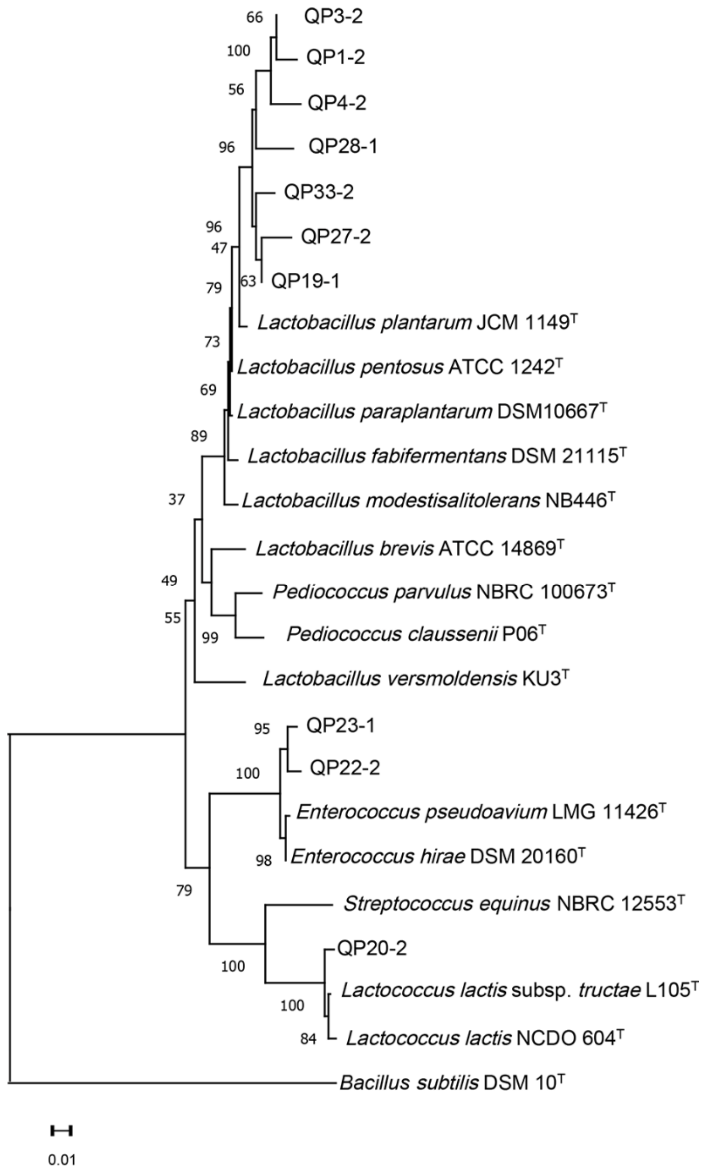

Figure S1. The phylogenetic tree of LAB strains isolated from Bamei pig feces based on 16S rRNA gene sequencing.

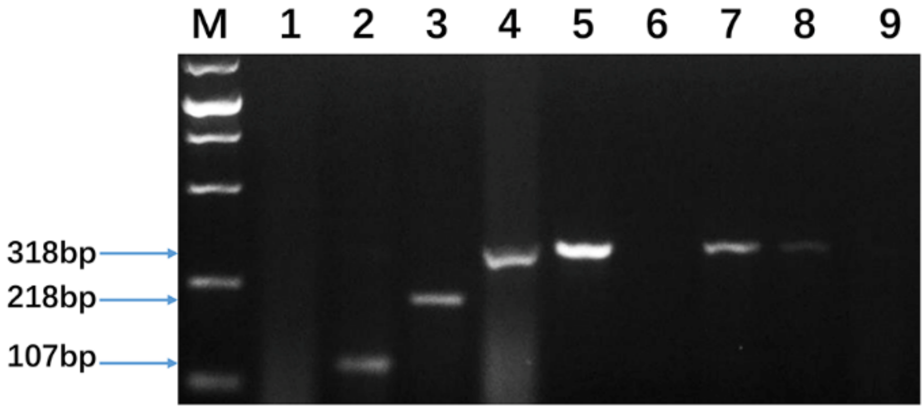

Figure S2. Amplification products obtained from the *rec*A multiplex assay. Lane M contains a

2000 bp PLUS DNA ladder. Lanes 1, 2, 3, and 4 refer to the PCR amplification products from *L. casei* JCM 16167^T (negative control), *L. paraplantarum* JCM 12533^T, *L. pentosus* JCM 1558^T, and *L. plantarum* subsp. *plantarum* JCM 1149^T, respectively; lanes 5, 6, 7, 8, and 9 refer to the PCR amplification product from LAB strains QP3-2, QP4-2, QP19-1, QP28-1, and QP33-2, respectively.

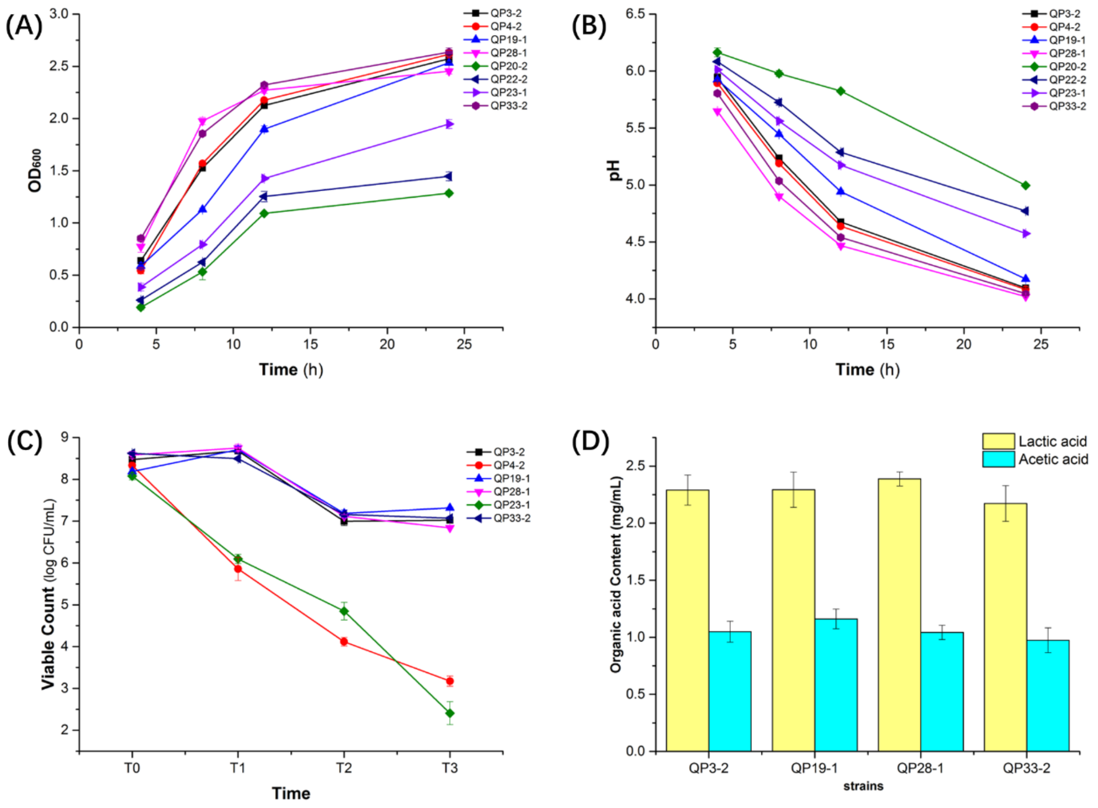

Figure S3. The results of the physiological and biochemical characteristics of the screened LAB. (A) Growth curve of eight LAB. (B) Acid production capacity of 8 selected LAB; (C) the viable counts (log CFU/mL) of 6 LAB strains under a simulated gastrointestinal tract (GIT) environment. Gastric juice T0 = the viability of LAB at the beginning of simulated gastric juice; gastric juice T1 = the viability of LAB under a simulated gastric environment for 3 hours; intestinal juice T2 = the viability at the beginning of intestinal juice; and intestinal juice T3 = the viability under simulation of intestinal juice for 4 hours. (D) The organic acid production of LAB strains QP3-2, QP19-1, QP28-1, and QP33-2.

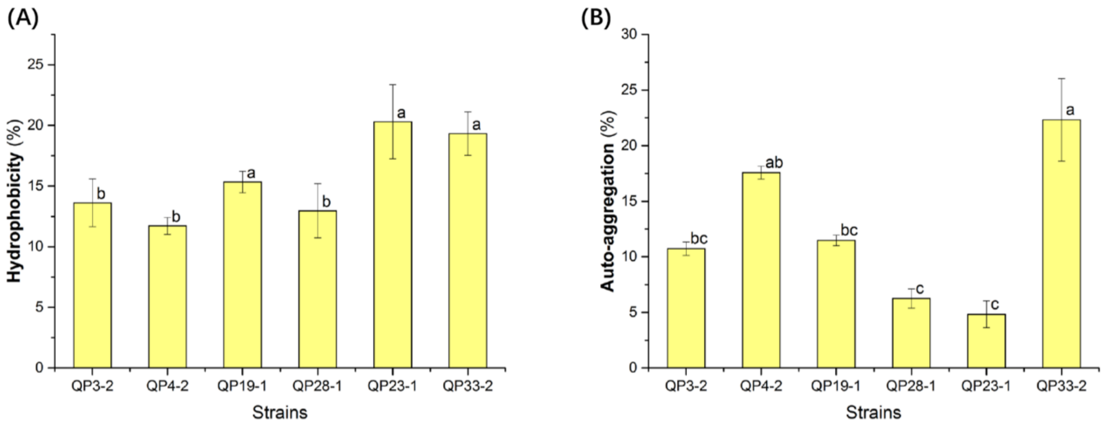

Figure S4. The surface hydrophobicity and auto-aggregation of the selected LAB strains. (A) The cell surface hydrophobicity (%) of LAB strains. (B) The auto-aggregation (%) of LAB strains. The results are expressed as the mean ± standard deviation (SD) (n=3).
